# Minimal force transmission between human thumb and index finger muscles under passive conditions

**DOI:** 10.1371/journal.pone.0212496

**Published:** 2019-02-15

**Authors:** Joanna Diong, Martin E. Héroux, Simon C. Gandevia, Robert D. Herbert

**Affiliations:** 1 School of Medical Sciences, Faculty of Medicine and Health, University of Sydney, Sydney, NSW, Australia; 2 Neuroscience Research Australia (NeuRA), Randwick, NSW, Australia; 3 University of New South Wales, Randwick, NSW, Australia; Universite de Nantes, FRANCE

## Abstract

It has been hypothesized that force can be transmitted between adjacent muscles. Intermuscle force transmission violates the assumption that muscles act in mechanical isolation, and implies that predictions from biomechanical models are in error due to mechanical interactions between muscles, but the functional relevance of intermuscle force transmission is unclear. To investigate intermuscle force transmission between human flexor pollicis longus and the index finger part of flexor digitorum profundus, we compared finger flexion force produced by passive thumb flexion after one of three conditioning protocols: passive thumb flexion-extension cycling, thumb flexion maximal voluntary contraction (MVC), and thumb extension stretch. Finger flexion force increased after all three conditions. Compared to passive thumb flexion-extension cycling, change in finger flexion force was less after thumb extension stretch (mean difference 0.028 N, 95% CI 0.005 to 0.051 N), but not after thumb flexion MVC (0.007 N, 95% CI -0.020 to 0.033 N). As muscle conditioning changed finger flexion force produced by passive thumb flexion, the change in force is likely due to intermuscle force transmission. Thus, intermuscle force transmission resulting from passive stretch of an adjacent muscle is probably small enough to be ignored.

## Introduction

When muscles produce force, it is thought that force is only transmitted in series from muscles to tendon and bone. This idea implies that muscles act in mechanical isolation and forms the basis for many animal and human biomechanical models [1]. However, mechanical connections (e.g. connective tissue) between adjacent muscles may transmit forces under physiological conditions [2–6]. Furthermore, it has been speculated that abnormal intermuscle force transmission may underlie pathological conditions such as spasticity and contracture [7]. Intermuscle force transmission violates the assumption that muscles act in mechanical isolation, and implies that predictions from many biomechanical models could be in error because of mechanical interactions between muscles. Consequently, it is important to quantify the size and direction of intermuscle force transmission in various conditions, including the passive stretch of an adjacent muscle.

One way to detect whether intermuscle force transmission is present is to measure changes in muscle force or length as an adjacent muscle is passively lengthened or actively stimulated. Many animal studies, especially those performed on rat leg muscles in situ, show that active muscle force can be transmitted laterally between muscles via myofascial networks when a single adjacent muscle is tetanically stimulated [8–10], or when synergistic or antagonistic muscles are tetanically stimulated [11, 12]. Changes of up to 30% of maximal active force are observed when adjacent muscles are stimulated [13]. In contrast, small changes of 0.8% of maximal active force are observed when an adjacent muscle is passively lengthened within physiological ranges of motion [12].

Another way to detect whether intermuscle force transmission is present is to measure the summation of muscle forces when adjacent muscles are stimulated in isolation or in combination. If muscles are not mechanically independent, muscle force produced by combined stimulation will be different to the sum of muscle forces produced by stimulation of the muscles in isolation. Experimentally, the differences in muscle force produced using this approach are less than 2% of maximal active force, on average [14]. This suggests the mechanical interactions are too small to invalidate the findings of biomechanical models that assume muscles act in isolation.

The magnitude of intermuscle force transmission is also variable in humans, shown by changes in muscle fascicle length and joint angle. In healthy people in vivo, moderate changes in soleus muscle length occur during passive knee extension (4.3 mm over 80° of knee extension; 1.2% of resting soleus length) and active stimulation of adjacent medial gastrocnemius (2.9 mm; 0.8% of resting soleus length [15]) [16]. In contrast, passive knee extension produces small changes in soleus muscle length (0.05 mm for each 1° change in ankle angle) [17]; when modeled using these estimates, intermuscle force transmission between gastrocnemius and soleus in healthy people is small [17]. Finally, increased passive tensioning in latissimus dorsi produces small changes in hip joint angle (0.8°) [18], which indicates that passive tension in an adjacent muscle generates sufficient force to induce joint angle changes in a large joint like the hip. It may be that intermuscle force transmisison occurs in humans but is small under physiological conditions [11] because differences in activation and contraction patterns between adjacent muscles abolish small amounts of lateral force transmission [19].

To assess the functional importance of intermuscle force transmission in human muscles by measuring changes in force, we investigated its size and direction at an anatomical site where lateral force transmission is likely to occur. The muscle bellies of flexor pollicis longus and flexor digitorum profundus lie adjacent to one another, and there is a connection between the tendons of flexor pollicis longus and the index finger part of flexor digitorum profundus in about one third of people [20, 21]. If intermuscle force transmission is present between these muscles via myofascial connections between the muscle bellies and possibly the tendinous connection, passive flexion of the thumb interphalangeal joint through full range of motion could cause flexion of the index finger joints through this or other links, especially the distal interphalangeal joint. Small amounts of active index finger flexion force are known to accompany small voluntary thumb flexion forces, but these are thought to be neurally-mediated effects, not mechanical interactions between muscles [22]. Therefore, we measured changes in index finger flexion force during passive thumb flexion. Small amounts of intermuscle force transmission are difficult to differentiate from movement artefact [23]. However it is possible to differentiate real effects from artefact by conditioning the muscles. Passive mechanical properties of muscles change in response to the history of previous muscle lengthening or contraction [24, 25]. If changes in the history of the long thumb flexor also change the magnitude of intermuscle force transmission, we can be confident that these effects are real because movement artefact is expected to be similar regardless of muscle history. Therefore, we also measured how intermuscle force transmission changes in response to either a prior thumb extension stretch or a prior thumb flexion maximal voluntary contraction (MVC). We hypothesized that there is intermuscle transmission of force between flexor pollicis longus and flexor digitorum profundus under passive conditions.

## Materials and methods

Fifteen healthy subjects participated in the study. Subjects were included if they were at least 18 years old and had no history of injury or surgery at the hand. The procedures conformed to the Declaration of Helsinki (2008) and were approved by the Human Research Ethics Committee of the University of New South Wales. All subjects gave informed consent.

**Fig 1:**
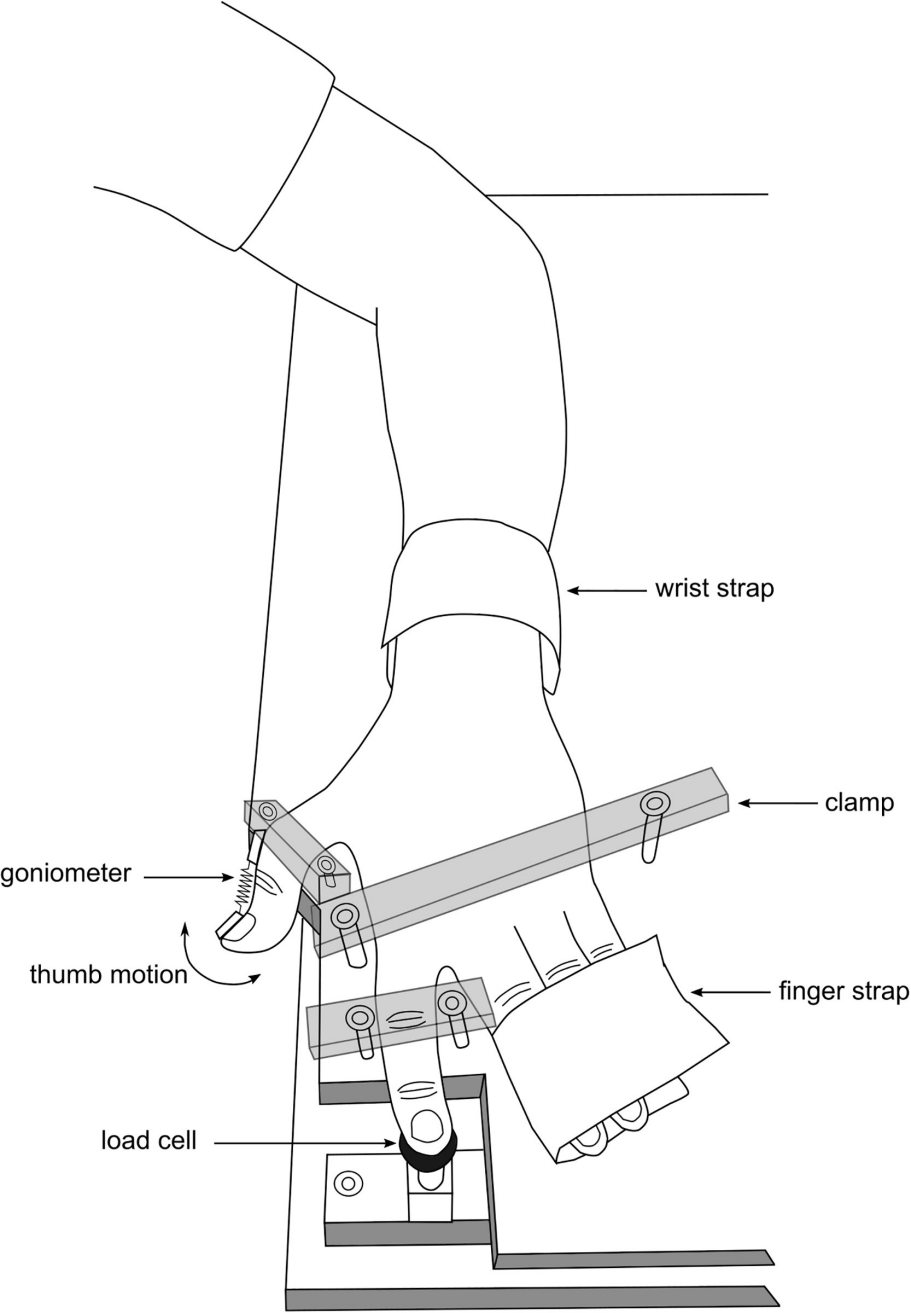
Experimental set-up. The forearm was supported in pronation. The thumb interphalangeal joint and index finger distal interphalangeal joint were free to move. Clamps and straps were used to stabalise the hand and prevent concomitant movement of any other finger or hand joint. An electrogoniometer measured the angle at the thumb interphalangeal joint, and a load cell measured force under the index finger. Electromyographic activity of the anterior forearm muscles was measured with surface electrodes (not shown).

Subjects were seated with the left arm supported in a custom built device (Fig 1). The forearm was supported and the hand was firmly stabilised with clamps and straps to allow free movement of the thumb interphalangeal joint and the index finger distal interphalangeal joint, but prevent movement of any other thumb, finger or hand joint. At the thumb carpometacarpal joint, the thumb was extended away from the fingers and firmly supported to maximise intermuscle force transmission between the thumb and index finger. A single axis electrogoniometer (Biometrics Single Axis Goniometer F35, Newport, UK) was attached to the distal and proximal thumb phalanges to measure changes in thumb interphalangeal joint range of motion. The distal phalanx of the index finger was positioned on a load cell that measured distal interphalangeal flexion force during finger flexion MVCs (Transducer Techniques MLP-50, California, USA) or passive thumb flexion and extension (Transducer Techniques MDB-5, California, USA; resolution 0.011 N). An adjustable post was used to stretch the thumb into extension and provide support during MVCs. A force-sensitive resistor attached to the post ensured that 6 N of force was used to stretch the thumb. Muscle activity was recorded with surface electromyography (EMG) electrodes (diameter 10 mm, interelectrode spacing 30 mm) placed over the muscle belly of flexor digitorum superficialis. Thumb angle signals were sampled at 50 Hz, index finger force signals were sampled at 500 Hz, and EMG signals were bandpass filtered at 10-500 Hz and sampled at 2000 Hz using Spike2 software with a 16-bit Cambridge Electronic Design 1401plus data acquisition board (CED, Cambridge, UK).

To investigate the effect of muscle conditioning on intermuscle force transmission, the long thumb flexor was conditioned in one of three ways prior to measuring index finger flexion force during passive thumb flexion: (i) six cycles of passive thumb flexion and extension, (ii) ten seconds of thumb interphalangeal extension stretch, to decrease stiffness in flexor pollicis longus [24], and (iii) a single thumb interphalangeal flexion MVC with the thumb interphalangeal joint in extension, to increase stiffness in flexor pollicis longus [24]. The flexion-extension cycling condition was used as the control condition to compare the stretch and MVC conditions because it simulated natural thumb movement. In response to the history of previous muscle lengthening or contraction, conditioning procedure (ii) was expected to decrease stiffness in flexor pollicis longus whereas conditioning procedure (iii) was expected to increase it [24].

Subjects first performed two index finger distal interphalangeal flexion MVCs against the load cell. Next, the removable post was adjusted to extend the thumb using 6 N, and the position of the post was marked before the thumb was released. Finally, the thumb interphalangeal joint was passively flexed and extended by an investigator to determine the limits of range of motion.

For each trial, the long thumb flexor was conditioned in one of three ways, before measuring index finger force as the thumb was passively flexed and extended by the investigator at an angular velocity of ∼5°/ s. The investigator followed a target trace to ensure the thumb was moved slowly. Three trials were collected for each of the conditioning protocols and trials were performed in random order. Subjects were instructed to relax during all trials.

There was little variation in force and angle measures across repeated trials, so data from the first trial for each condition were used in the analysis. Index finger force and thumb angle signals were digitally low-pass filtered at 10 Hz (dual-pass, 4th order Butterworth filter), and force signals were downsampled to 50 Hz. Forearm flexor muscle EMG signals were digitally bandpass filtered at 20-450 Hz (dual-pass, 4th order Butterworth filter). It was important to ensure that changes in index finger force during passive thumb movement were not due to movement artefact. Since movement artefact was more likely to occur at the extremes of thumb range of motion, only data from the central portion of thumb range of motion were used in the analysis: when the thumb was flexed from 25° extension to 25° flexion, where 0° indicates the thumb was in its neutral position. For each subject, slopes from linear regression of index finger force and thumb angle quantified the change in index finger force for a 1° change in passive thumb motion.

**Fig 2:**
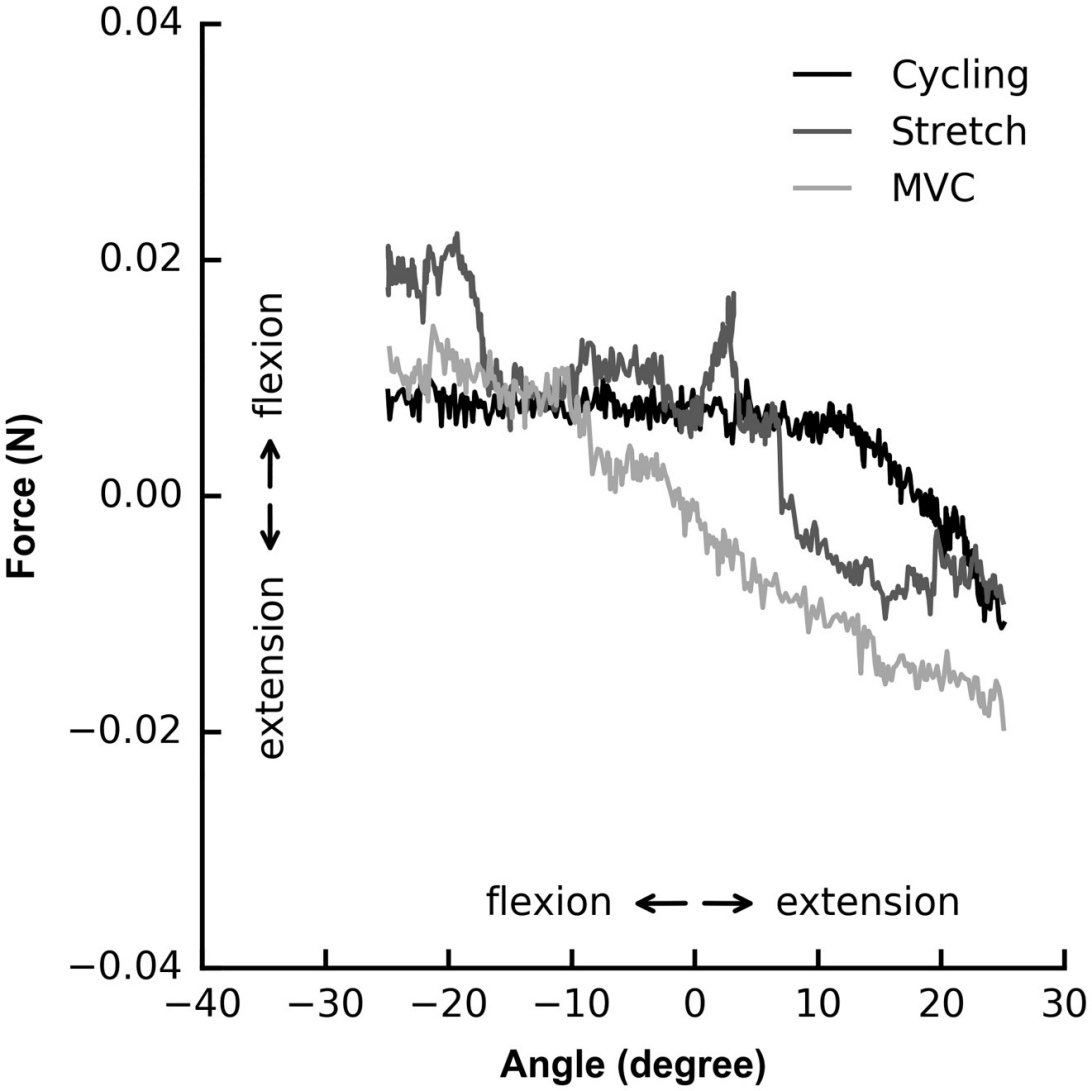
Index finger force as the thumb is moved from an extended to a flexed position for one subject. Data show change in force after passive thumb flexion flexion-extension cycling, thumb extension stretch, or thumb flexion MVC. Sign conventions for flexion and extension directions of angle and force are shown.

**Fig 3:**
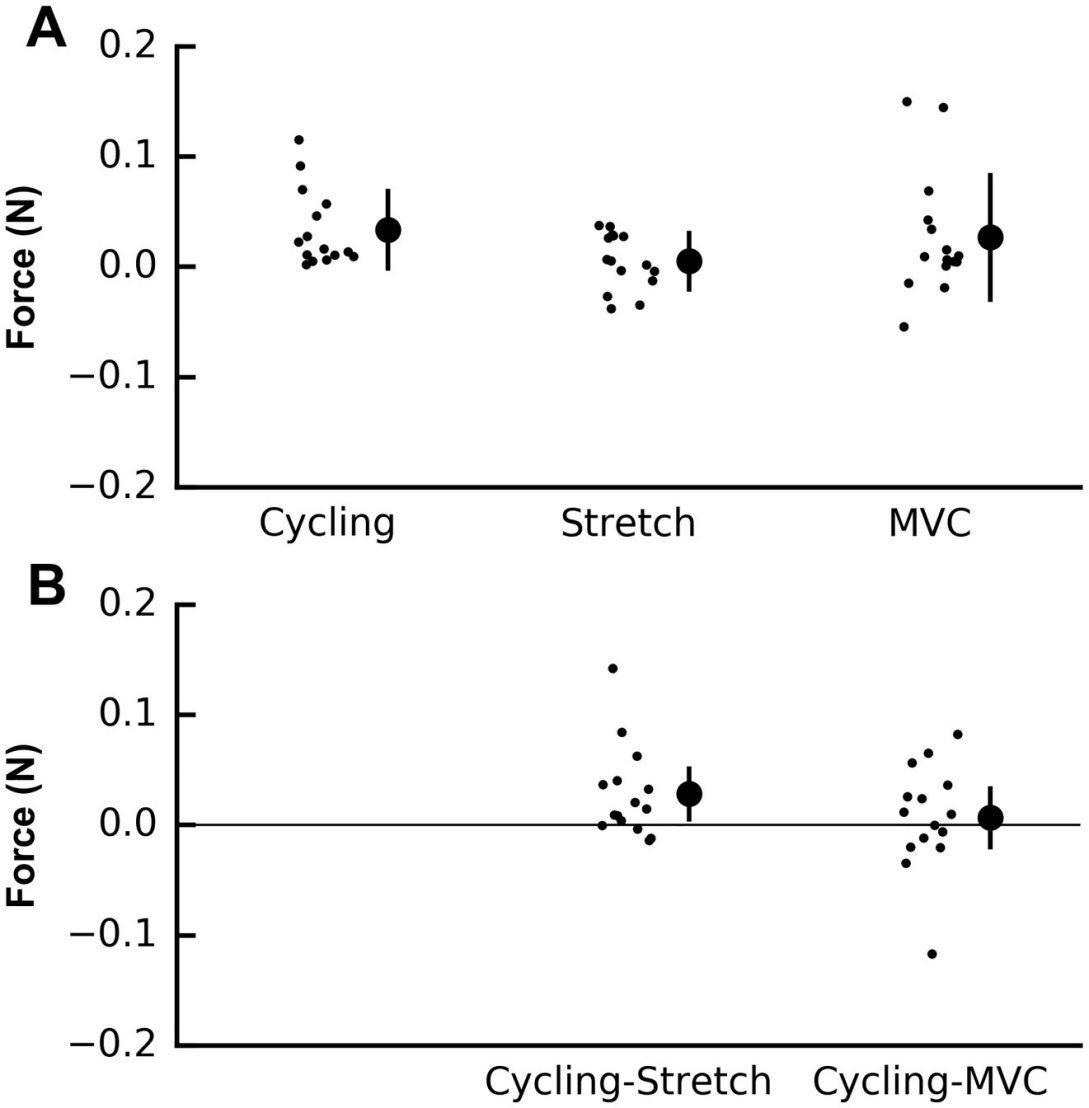
Within- and between-condition change in index finger force. (A) Individual subject data and mean ± SD of index finger force after passive thumb flexion flexion-extension cycling, thumb extension stretch, and thumb flexion MVC for the 15 subjects. Each data point is the change in index finger force for a 1° change in passive thumb motion for that subject. (B) Individual subject data and mean difference (95% CI) of index finger force after thumb extension stretch or after thumb flexion MVC, compared to thumb flexion-extension cycling. Each data point is the between-condition paired difference of the change in index finger force for a 1° change in passive thumb motion for that subject.

Changes in index finger flexion force during passive thumb flexion were described with means and standard deviations (SD), and were compared between the control condition and the stretch and MVC conditions by calculating paired differences in force for each subject. The t-distribution was used to calculate the mean and 95% confidence intervals (95% CI) of these differences. Data and code used to generate Figs 2 and 3 are available in the supporting information.

## Results

Data were collected from 15 healthy subjects (data are reported as mean ± SD unless otherwise stated: age 44 ± 11 years; 7 male, 8 female). On average, index finger flexion MVC force was 32 ± 14 N. Subjects remained relaxed during testing; average EMG amplitude was 1.8 ± 1.3%MVC during cycling trials, 2.6 ± 1.8%MVC during stretch trials, and 2.9 ± 2.0%MVC during MVC trials. Passive thumb angular velocity was 6.3 ± 1.6°/ s (max 9.5°/ s) during cycling trials, 4.6 ± 1.2°/ s (max 7.3°/ s) during stretch trials, and 4.9 ± 1.9°/ s (max 8.9°/ s) during MVC trials. Data for one subject (Fig 2) and summary data for all subjects (Fig 3) are shown.

Index finger flexion force during passive thumb flexion increased after all three long thumb flexor conditioning protocols. As the thumb was passively flexed from 25° extension to 25° flexion, on average, index finger flexion force increased by 0.034 ± 0.035 N after passive thumb flexion-extension cycling, by 0.005 ± 0.025 N after thumb extension stretch, and by 0.027 ± 0.056 N after thumb flexion MVC (Fig 3A). These forces correspond to 0 to 0.1% of MVC index finger flexion force.

Comparing between conditioning protocols, intermuscle force transmission was present between flexor pollicis longus and flexor digitorum profundus to the index finger. The increase in finger flexion force was less after conditioning with thumb extension stretch than after conditioning with passive thumb flexion-extension cycling (mean difference between conditions: 0.028 N, 95% CI 0.005 to 0.051 N, *t*_14_ = 2.54, p = 0.020; Fig 3B). The mean increase in finger flexion force after thumb extension stretch corresponds to 0.1% of MVC index finger flexion force. In contrast, there was no difference between conditioning with thumb flexion MVC and passive thumb flexion-extension cycling (0.007 N, 95% CI -0.020 to 0.033 N, *t*_14_ = 0.53, p = 0.59; Fig 3B).

## Discussion

This is the first study to measure the size and direction of changes in force caused by passive intermuscle force transmission between the thumb and index finger in humans. We obtained a direct measure of muscle output (i.e. force) rather than an indirect measure such as change in muscle fascicle length or muscle displacement. Overall, index finger flexion force increased during passive thumb flexion after the long thumb flexor was conditioned in one of three ways. Small amounts of intermuscle force transmission, such as those that might occur when an adjacent muscle is passively stretched, are difficult to differentiate from movement artefact [23]. In this study, we can be confident that the small amounts of intermuscle force transmission observed are real because they could be manipulated by changing the history of previous muscle lengthening.

This study extends previous work on intermuscle force transmission in humans by examining force transmission at the hand. Small amounts of index finger flexion force accompany active thumb flexion during grasping [22]. However it is unclear whether finger flexion force would likewise accompany passive thumb flexion. Our findings are consistent with previous reports of small changes in muscle fascicle and muscle-tendon length due to intermuscle force transmission in healthy people or in people with stroke who had contracture [16, 23]. Negligible amounts of intermuscle force transmission were also predicted by biomechanical models of human muscles [17].

We found that small forces (0.005 to 0.034 N) were transmitted between the thumb and index finger. Schuind and colleagues measured force in the flexor pollicis longus muscle in situ during thumb interphalangeal flexion-extension and reported that the mean passive force was 2.9 N [26]. Based on these data, we estimate that intermuscle force transmission corresponds to 0.17 to 1.17% of passive flexor pollicis longus force. Under active conditions, the product of flexor pollicis longus physiological cross-sectional area (2.10 cm^2^, [27]) and muscle stress (approximately 30 N cm^−2^, [28]) gives a conservative estimate of 63 N for maximal isometric muscle force. Here, intermuscle force transmission corresponds to ∼0.05% of maximal active flexor pollicis longus force. Even if we discount the small effects during active conditions and restrict our conclusions to passive conditions, intermuscle force transmission is still small.

Our findings need to be interpreted in context of methodological limitations. First, passive force in flexor pollicis longus in vivo was not known and cannot be directly measured in humans except during invasive surgical procedures. If force in flexor pollicis longus was small, then intermuscle force transmission between flexor pollicis longus and flexor digitorum profundus to the index finger would be even smaller and harder to detect. Although we found only small amounts of intermuscle force transmission, muscle activity in superficial extensor digitorum and deep extensor indicis may have decreased net index finger flexion force. Similarly, activity in the deep flexor digitorum profundus may have contributed to index finger force. Unfortunately, activity in these muscles was not recorded. We investigated the size and direction of lateral force transmission at an anatomical site where it is likely to occur, but it is not known how many subjects had the anatomical connection between the tendons of those muscles. Regardless of whether this anatomical connection was present, the bellies of the two investigated muscles lie adjacent to each other and are connected to each other via connective tissue. These connections could transmit forces between muscles, as has been shown in previous studies on other muscle pairs. Lastly, the resolution of the load cell (0.011 N) was close to the range of effects detected (mean difference between cycling-stretch conditions: 0.028 N), so the load cell may not have detected differences in intermuscle force transmission between the other pair of conditions.

Methodological limitations may also have contributed to why stretching the long thumb flexor decreased intermuscle force transmission, whereas maximally contracting the same muscle did not. If passive thumb motion was systematically faster or slower between conditions, thumb motion angular velocity may have produced systematic effects of intermuscle force transmission. To prevent this, the investigator followed a target trace to move the thumb slowly. On average, thumb motion was slow, and maximal angular velocities never exceeded those typically applied in studies on passive movement [29]. Overall however, thumb motion was slightly faster during cycling compared to either stretch or MVC trials. This implies that faster thumb motion during cycling may have caused larger intermuscle force transmission compared to stretch. However, this cannot explain why faster thumb motion during cycling did not similarly cause larger intermuscle force transmission compared to MVC. Not knowing how thumb motion influences index finger force or motion of flexor pollicis longus is a limitation of this study.

Linear regression was used to quantify change in index finger force for a 1° change in passive thumb motion, but the regression residuals in time-series data are not random or statistically independent. The intention was to estimate, but not to infer the change in finger force during thumb motion for each trial, so we did not calculate 95% CI about the slope for each trial. We chose to perform linear regression as the simplest procedure to estimate an average change in force over the range of thumb angles and avoid complex non-linear fits. In the analysis plan, we pre-specified the key comparisons of interest as the differences in intermuscle force transmission between the control-stretch and control-MVC conditions, and present mean differences and 95% CI so readers may infer the precision of effects. We did this to reduce the number of statistical comparisons and avoid conducting post-hoc tests which generate p-values that are conditional on p-values [30, 31]. Accordingly, we found small differences between one pair of conditions but not another.

Stretching and maximally contracting muscle to change the history of muscle lengthening are routinely used to examine proprioceptive outcomes [25, 32]. The small decrease in intermuscle force transmission after thumb extension stretch might result from viscous deformation of flexor pollicis longus during the stretch, or stretch-induced strain of myofascial connections or the tendinous connection. These mechanisms would decrease stiffness of flexor pollicis longus and thus, presumably, transfer less force laterally to the index finger part of flexor digitorum profundus. We did not observe a corresponding change in intermuscle force transmission after maximally contracting the long thumb flexor at a long muscle length because this effect may have been too small to detect. For our sample, the mean increase in finger flexion force during passive thumb flexion was smaller after conditioning with either thumb extension stretch and thumb flexion MVC, compared to passive thumb flexion-extension cycling. The 95% CI of the cycling-MVC mean difference crossed 0 N, indicating the absence of an effect. In contrast, the 95% CI of the cycling-stretch mean difference did not cross 0 N, indicating this form of conditioning did have an effect on passive force transmission. Importantly, the width of these CI were narrow, indicating these estimates were precise, especially for magnitudes of forces ∼1% of passive force in flexor pollicis longus. Even if the effect of maximally contracting the long thumb flexor was too small to detect, it is arguable whether such small amounts of intermuscle force tranmission are physiologically relevant or worthwhile.

## Conclusion

In summary, these findings indicate that intermuscle force transmission occurs between the thumb and index finger muscles under passive physiological conditions. But these amounts of lateral force transmission are small and probably of little functional relevance.

## Supporting information

S1 File Data and code. Comma-separated-values (CSV) data files and Python file to generate Figs 2 and 3. See the README.txt file for a full description.
(ZIP)
