# Supplementary figures and images for: Minimal force transmission between human thumb and index finger muscles under passive conditions
Source: PLoS One. 2019 Feb 15;14(2):e0212496. doi: 10.1371/journal.pone.0212496 (PMC6377133; doi:10.1371/journal.pone.0212496)

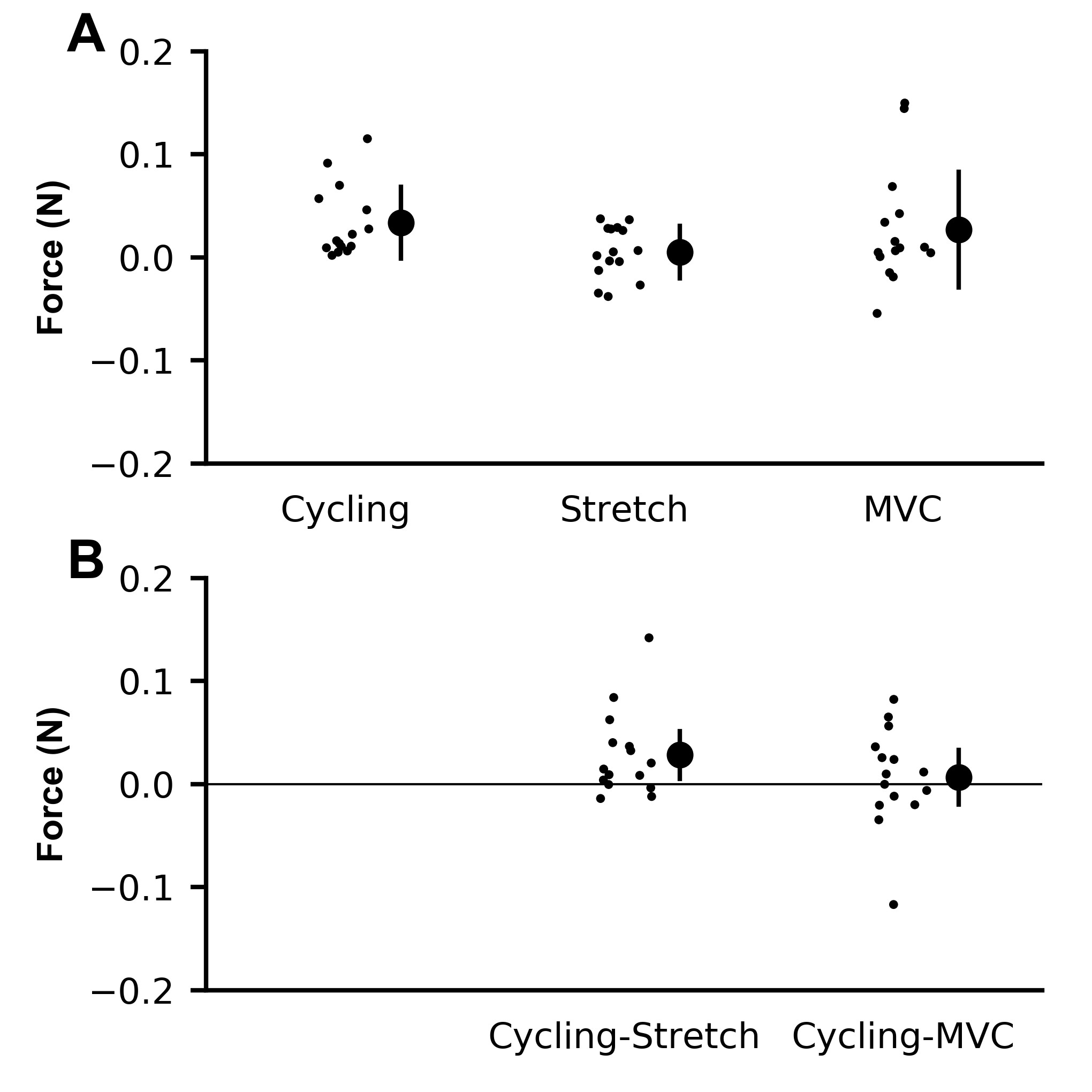

Supplement: S1 File — Comma-separated-values (CSV) data files and Python file to generate Figs 2 and 3. See the README.txt file for a full description. (ZIP) [file pone.0212496.s001.zip › S1 File/Fig3.tiff]

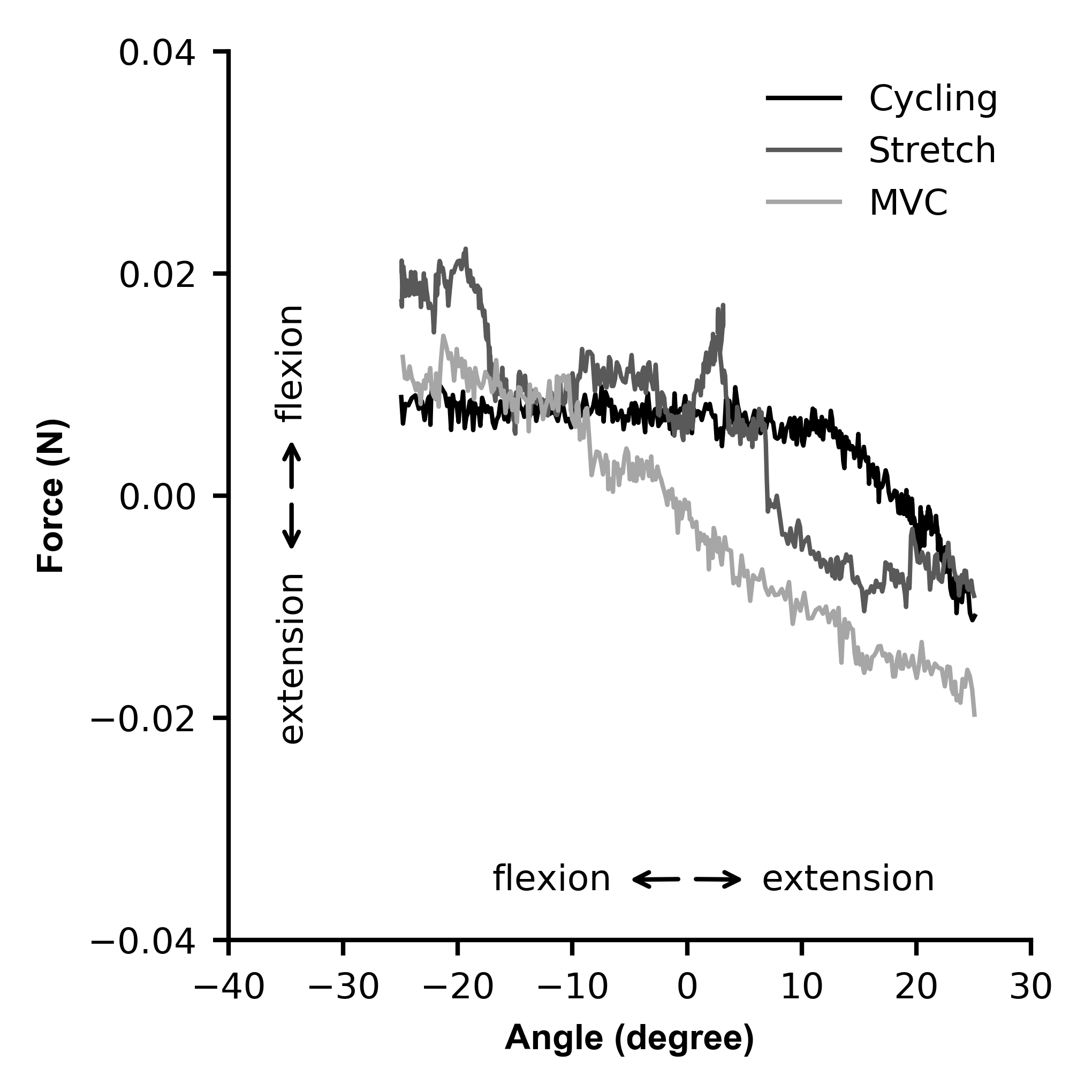

Supplement: S1 File — Comma-separated-values (CSV) data files and Python file to generate Figs 2 and 3. See the README.txt file for a full description. (ZIP) [file pone.0212496.s001.zip › S1 File/Fig2.tiff]
